# Irreversible Electroporation for Liver Metastases from Colorectal Cancer: A Systematic Review

**DOI:** 10.3390/cancers15092428

**Published:** 2023-04-24

**Authors:** Harry V. M. Spiers, Francesco Lancellotti, Nicola de Liguori Carino, Sanjay Pandanaboyana, Adam E. Frampton, Santhalingam Jegatheeswaran, Vinotha Nadarajah, Ajith K. Siriwardena

**Affiliations:** 1 Cambridge Hepato-Pancreato-Biliary Unit, Addenbrooke’s Hospital, Cambridge CB2 0QQ, UK; harryspiers@doctors.org.uk; 2 Department of Surgery, University of Cambridge, Cambridge CB2 0QQ, UK; 3 Hepato-Pancreato-Biliary Unit, Manchester Royal Infirmary, Manchester M13 9WL, UK; md.francesco.lancellotti@gmail.com (F.L.);; 4 HPB and Transplant Unit, Freeman Hospital, Newcastle-upon-Tyne NE7 7DN, UK; 5 Hepato-Pancreato-Biliary Surgery Unit, Royal Surrey NHS Foundation Trust, Guildford GU2 7XX, UK; adam.frampton@surrey.ac.uk; 6 Section of Oncology, Deptartment of Clinical & Experimental Medicine, University of Surrey, Guildford GU2 7WG, UK; 7 Department of Radiology, Manchester Royal Infirmary, Manchester M13 9WL, UK; vinotha.nadarajah@mft.nhs.uk

**Keywords:** colorectal cancer, liver metastases, ablation, irreversible electroporation, systematic review

## Abstract

**Simple Summary:**

This is a systematic review of irreversible electroporation (IRE) of colorectal hepatic metastases. The results show that IRE is associated with low procedure-related morbidity and mortality. Disease-specific data on the indications and outcome of IRE as a treatment for patients with colorectal hepatic metastases are limited as are comparative data. More prospective studies are required to define the role of IRE in the portfolio of treatments for patients with liver metastases from colorectal cancer.

**Abstract:**

Background: Irreversible electroporation (IRE) is a non-thermal form of ablation based on the delivery of pulsed electrical fields. It has been used to treat liver lesions, particularly those in proximity to major hepatic vasculature. The role of this technique in the portfolio of treatments for colorectal hepatic metastases has not been clearly defined. This study undertakes a systematic review of IRE for treatment of colorectal hepatic metastases. Methods: The study protocol was registered with the PROSPERO register of systematic reviews (CRD42022332866) and reports in compliance with the preferred reporting items for systematic reviews and meta-analyses (PRISMA). The Ovid MEDLINE^®^, EMBASE, Web of Science and Cochrane databases were queried in April 2022. The search terms ‘irreversible electroporation’, ‘colon cancer’, ‘rectum cancer’ and ‘liver metastases’ were used in combinations. Studies were included if they provided information on the use of IRE for patients with colorectal hepatic metastases and reported procedure and disease-specific outcomes. The searches returned 647 unique articles and the exclusions left a total of eight articles. These were assessed for bias using the methodological index for nonrandomized studies (MINORS criteria) and reported using the synthesis without meta-analysis guideline (SWiM). Results: One hundred eighty patients underwent treatment for liver metastases from colorectal cancer. The median transverse diameter of tumours treated by IRE was <3 cm. Ninety-four (52%) tumours were adjacent to major hepatic inflow/outflow structures or the vena cava. IRE was undertaken under general anaesthesia with cardiac cycle synchronisation and with the use of either CT or ultrasound for lesion localisation. Probe spacing was less than 3.2 cm for all ablations. There were two (1.1%) procedure-related deaths in 180 patients. There was one (0.5%) post-operative haemorrhage requiring laparotomy, one (0.5%) bile leak, five (2.8%) post-procedure biliary strictures and a zero incidence of post-IRE liver failure. Conclusions: This systematic review shows that IRE for colorectal liver metastases can be accomplished with low procedure-related morbidity and mortality. Further prospective study is required to assess the role of IRE in the portfolio of treatments for patients with liver metastases from colorectal cancer.

## 1. Introduction

In 2020, the European commission estimated that colorectal cancer accounted for 12.7% of all new cancer diagnoses and 12.4% of all deaths due to cancer, making it the second most frequently occurring cancer [1]. About one-fifth of patients with colorectal cancer will have liver metastases at the time of presentation [2]. Hepatic metastases may also be detected during the disease course [2]. Current standards of care for patients with colorectal cancer liver metastases and performance metrics that permit active treatment are realized through a combination of systemic chemotherapy (with radiotherapy for selected patients with rectal primary tumours) and surgery to remove the disease from both the primary and liver metastatic sites [3,4]. If resection is not feasible, ablation of liver tumours is possible and has been evaluated such that the EORTC 4004 CLOCC study reported a 30-month overall survival (OS) of 61.7% (95% confidence interval (CI) 48.2–73.9%) for patients treated by radiofrequency ablation in addition to systemic chemotherapy versus 57.6% (95% CI 44.1–70.4%) in the chemotherapy alone group [5]. After a median follow up of 9.7 years, OS was significantly improved in the radiofrequency plus chemotherapy group (HR = 0.58; 95% CI 0.38–0.88) with an eight-year OS of 35.9 versus 8.9% for chemotherapy alone. Radiofrequency and microwave are forms of thermal ablation and are thus limited in their application near major vessels within the liver, as blood flow conducts thermal energy away from the tumour creating a “heatsink” effect [6].

Irreversible electroporation (IRE) is a process that deliver pulses of electrical current of high voltage between electrodes [7]. The technique has been shown to cause cell death within an ablation zone in the liver between these electrodes [8]. An initial study (COLDFIRE-1) established that IRE, applied two hours prior to resection, was associated with cell death and necrosis of liver metastases of colorectal cancer origin [9]. A phase II study (COLDFIRE-2) showed that in a series of 51 patients with colorectal liver metastases of 5.0 cm or smaller, IRE ablation was successful in 50 patients (98%) with local control at twelve months in 74% [10]. Procedure-related morbidity was seen in 23 participants with one fatality. 

Although IRE has been used to treat a range of both primary and metastatic liver tumours the current place of the technique in the portfolio of treatments for colorectal hepatic metastases is has not been established. Although there appears to be promising anti-tumour activity, it is not clear whether this technique complements or replaces thermal ablation. Further, it remains to be established whether IRE can be used in conjunction with surgical resection and also whether IRE can be combined with external radiotherapy.

This study focuses on the use of IRE to treat liver metastases of colorectal origin. The objective of this systematic review is to assess the current reported use, safety profile and outcome of IRE for colorectal hepatic metastases.

## 2. Methods

### 2.1. Design

This is a systematic review of the use of irreversible electroporation to treat patients with colorectal hepatic metastases. This review is reported in compliance with the preferred reporting items for systematic reviews and meta-analyses (PRISMA) checklist [11].

### 2.2. Protocol and Registration

The study protocol was registered with the PROSPERO register of systematic reviews (CRD42022332866). 

### 2.3. Information Sources

The Ovid MEDLINE^®^, EMBASE, Web of Science and Cochrane databases were queried in April 2022. The search terms ‘irreversible electroporation’, ‘colon cancer’, ‘rectum cancer’ and ‘liver metastases’ were used in combination (see Supplementary Table S1 for full search strategy).

### 2.4. Eligibility Criteria

Studies were included if they provided information on the use of IRE for patients with colorectal hepatic metastases and reported disease- and procedure-specific outcomes. Publications that included the use of IRE for liver tumours that did not distinguish between colorectal liver metastases and other tumour types were excluded. Case reports, studies reporting less than five patients, as well as those not published in English, opinion pieces, review articles and guidelines on the use of IRE for colorectal hepatic metastases were also excluded.

### 2.5. Search Strategy and Study Selection

**Figure 1:**
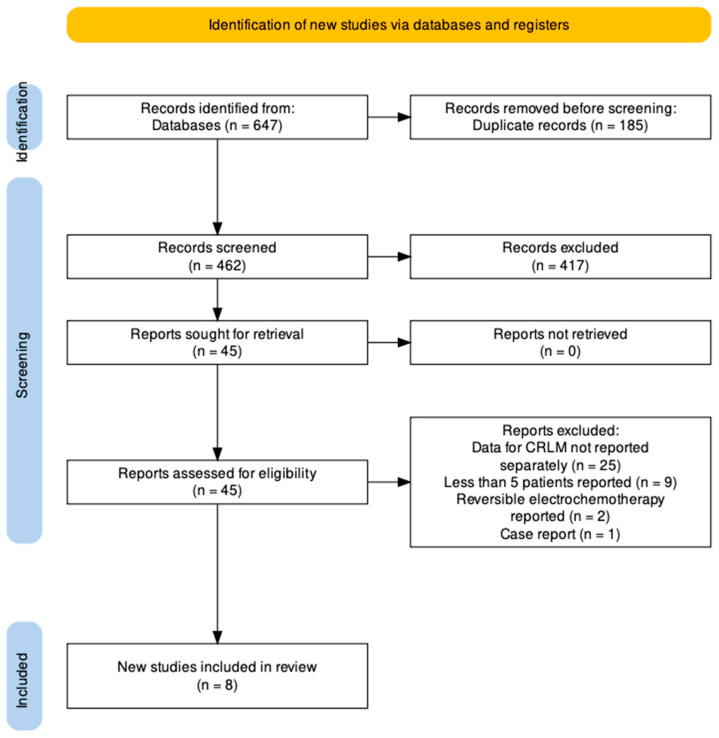
PRISMA Flowchart.

Details of the search are seen in the PRISMA flowchart (Figure 1). Searches returned 647 unique articles. Reports were independently screened by two authors (HVMS and FL). After exclusion of 185 duplicates and 417 records which met initial exclusion criteria, 45 reports were sought for full text review. Twenty-five studies were excluded as they reported the role of IRE in liver tumours without separate description of outcomes in patients with colorectal hepatic metastases. Nine studies reported less than five patients and were excluded. Two studies reported on electrochemotherapy with reversible electroporation and were excluded. One case report was also excluded. This left a total of eight studies which constitutes the study population of this report.

### 2.6. Assessment for Bias

A methodological index for nonrandomized studies was used [12]. MINORS items for adequacy of the control group, use of contemporary control groups, baseline equivalence of groups and adequate statistical analyses could not be assessed for the reports in this study.

### 2.7. Extraction of Data

Data were extracted based on type of study, demographic and disease profile together with information on tumour characteristics and interventions prior to IRE. Details on location of tumours in relation to major hepatic vasculature was sought. Information on technical details of the use of IRE, including number and hepatic location of lesions, was sought. Treatment-related morbidity in relation to post-procedure bile leak, haemorrhage and liver failure were sought together with procedure-related mortality, time to recurrence and both progression-free and overall survival.

### 2.8. Synthesis of Results

Study heterogeneity precluded meta-analysis, thus synthesis without meta-analysis (SWiM) in systematic reviews was used to structure this report [13]. All the studies included in this report describe the use of IRE for patients with colorectal liver metastases. There are no standardised metrics for reporting. Data assembly followed the clinical pathway from patient and disease demographic profile, prior intervention, to use of IRE and outcome. 

### 2.9. Ethics Review

The NHS Health Research Authority questionnaire deemed that this study was not research as the participants are not randomized to different groups, no individual-specific reporting is undertaken, there is no change in treatment or patient care and the findings cannot be regarded as wholly generalizable [14].

### 2.10. Role of the Manufacturer in Production of the Report

The manufacturer had no role in the concept, design, execution or writing of the report.

## 3. Results

### 3.1. Methodological Index for Non-Randomized Studies (MINORS) Scores (Table 1) [9,10,15,16,17,18,19,20]

The median (range) MINORS score was 11 (8–15) with a maximum of 15. It can be seen that an unbiased evaluation of endpoints was not provided by any of the studies in this series and one study provided a prospective calculation of sample size.

**Table 1:** Methodological index for non-randomized studies (MINORS) scores.

Author (Year)	MINORS Criteria Assessed								
	Clearly Stated Aim	Consecutive Patients	Prospective Collection of Data	Endpoint Appropriate to The Study Aim	Unbiased Evaluation of Endpoints	Follow-Up Period Appropriate to the Major Endpoint	Loss to Follow Up Not Exceeding 5%	Prospective Calculation of Study Size	Overall MINORS Score
Hosein PJ (2014) [15]	1	1	2	2	1	2	2	0	11
Scheffer HJ (2014) [9]	2	2	2	2	1	2	2	0	13
Eller A (2015) [16]	2	0	1	2	1	2	1	0	9
Beyer L P (2017) [17]	1	1	0	1	1	2	2	0	8
Frühling P (2017) [18]	2	2	2	1	1	2	2	0	12
Cornelis FH (2020) [19]	2	2	0	2	1	2	2	0	11
Meijerink M (2021) [10]	2	2	2	2	1	2	2	2	15
Hitpass L (2022) [20]	2	2	0	2	1	2	2	0	11

### 3.2. Study Type and Demographic Profile (Table 2)

Of the eight studies included in this report, five were prospective Phase II studies and three were retrospective case cohorts. A total of 217 patients were included of whom 180 underwent treatment for liver metastases from colorectal cancer. The median (range) number of patients per report was 21 (8–51). The largest single report in this series included 51 patients. Sidedness of primary tumour was reported in three studies (38%) and presence/absence of extrahepatic disease was specifically reported in two studies (25%).

**Table 2:** Study type and demographic profile.

Author (Year)	Study Type	Total Number of Patients	Patients with CRLM	Median (Range) Age in Years	Female (%)	Primary Right:Left	Extrahepatic Disease
Hosein PJ (2014) [15]	retrospective observational	29	29	62 (32–83)	12 (41)	n/a	3 (10%)
Scheffer HJ (2014) [9] *	Prospective cohort	10	10	63 (49–74)	6 (60)	5:5	n/a
Eller A (2015) [16]	Prospective Phase II	14	8	60 (36–73)	0 (0)	n/a	n/a
Beyer L P (2017) [17]	Retrospective Observational	35	18	60(46–78) (all cohort)	n/a	n/a	n/a
Frühling P (2017) [18]	Prospective Phase II	30	16	66 (56–78)	4 (25)	n/a	n/a
Cornelis FH (2020) [19]	Retrospective observational	25	25	n/a	12 (48)	n/a	n/a
Meijerink M (2021) [10]	Prospective Phase II	51	51	67 (39–82)	14 (28)	13:38	n/a
Hitpass L (2022) [20]	Prospective cohort	23	23	60 ± 11 (mean, sd)	8 (35%)	5:18	0 **
Total		217	180				

CRLM = colorectal liver metastases. n/a = not available. Note that “left” sided tumours also include rectal tumours. * Patients underwent IRE followed by liver resection of the ablated tumour. ** Extrahepatic disease termed “no prognostically relevant disease”.

### 3.3. Tumour Characteristic and Interventions Prior to IRE (Table 3)

A total of 315 tumours were treated in 180 patients. No studies reported the use of IRE to treat tumours with a median transverse diameter of >3 cm. Five reports provided anatomical localization detail indicating that IRE was utilised for patients with tumours which were either close to hepatic inflow, outflow or vena cava. Three studies reported the use of prior chemotherapy and indicate that this was used between 65 and 100% of patients prior to IRE. One study provided information on surgical treatment of the primary tumour. Four studies included patients treated by prior hepatic resection. Use of other ablative modalities was reported by three studies. Other treatments included radiofrequency ablation, thermal ablation (non-specified), trans-arterial chemo-embolization and selective internal radiation therapy (SIRT).

**Table 3:** Tumour characteristics and interventions prior to IRE.

Author (Year)	Median Number of Tumours (Range)	Total Number of Tumours	Median (Range) Transverse Size in cm	Inflow/ Outflow Proximity	Interventions Prior to IRE			
					Chemo Therapy	Surgery to Primary	Surgery to Metastases	Other Treatments
Hosein PJ (2014) [15]	2 (1–6)	58	2.7 (1.2–7.0)	11 IVC, 9 PV, 8 HV, 3 HA	29 (100)	0	13 (45)	2 RFA, 1 TACE, 2 SIRT
Scheffer HJ (2014) [9]	n/a	54	2.4 (0.8–5.3)	n/a-	8 (80)	n/a-	10	30 thermal ablations. 14 resections
Eller A (2015) [16]	1 (1–2)	11	1.8 (0.8–2.3)	3 RHV, 2 IVC, 4 MHV, 2 LPV, LHV, RPV	n/a	n/a	n/a	n/a-
Beyer L P (2017) [17]	n/a	n/a	2.5 ± 1.1	5 RPV 5 LPV 4 HV 4 IVC	n/a	n/a	n/a	n/a
Frühling P (2017) [18]	1 (1–2)	23	2.4 (0.8–1.4)	n/a	n/a	n/a	16 (100%)-	n/a
Cornelis FH (2020) [19]	n/a	29	2.1	Portal or HV	n/a	n/a	n/a	n/a
Meijerink M (2021) [10]	1 (1–4)	76	2.2 (0.5–5.4)	n/a	33 (65%)	n/a	13 (26)	4 Thermal ablation; 2 SBRT
Hitpass L (2022) [20]	n/a	32	1.5 (0.4–3.9)	13 LPV, 5 RPV, 10 MHV, 2 RHV, 2 IVC	n/a	n/a	23 (100%)	n/a
Total		283						

IVC = inferior vena cava; PV = portal vein; LPV = Left portal vein; RPV = Right portal vein; HV = Hepatic veins; MHV = middle hepatic vein; RHV = Right hepatic vein; LHV = Left Hepatic vein; HA = hepatic artery; RFA = radiofrequency ablation; TACE = Trans-arterial chemo-embolisation; SIRT = Selective internal radiation therapy; SBRT = Stereotactic body radiation therapy.

### 3.4. Details of IRE Procedures (Table 4)

Two hundred eighty-three colorectal liver metastases were treated by IRE in 162 patients in whom these data were available (average of 1.8 lesions per patient). In all reports, IRE was undertaken under general anaesthesia with cardiac cycle synchronisation and with the use of either CT or ultrasound for lesion localisation. Probe spacing was less than 3.2 cm for all ablations.

**Table 4:** Details of IRE procedures.

First Author (Year)	IRE Model	Method of Probe Placement	Type of Anaesthesia	Route	Pulse Synchro	Probe Spacing (cm)	Treatment Pulses	Ablations
Hosein P 2014 [15]	NanoKnife^®^, AngioDynamics	CT	GA	Perc	Yes	1.1–2.4	n/a	36 (total)
Scheffer HJ 2014 [9]	NanoKnife^®^, AngioDynamics	USS	GA	Open	Yes	<2.5	90	10
Eller A 2015 [16]	NanoKnife^®^, AngioDynamics	CT	GA	Perc	Yes	<2	90	12
Beyer LP 2017 [17]	NanoKnife^®^, AngioDynamics	CT	GA	Perc	n/a	3.1 (average)	n/a	n/a
Frühling P 2017 [18]	NanoKnife^®^, AngioDynamics	CT	GA	Perc	Yes	<2	90	n/a
Cornelis FH 2020 [19]	NanoKnife^®^, AngioDynamics	CT	GA	Perc	Yes	1.5 (1–2.5)	90 (70–90)	n/a
Meijerink (2021) [10]	NanoKnife^®^, AngioDynamics	CT—perc USS—open	GA	Perc Open	Yes	<2.5	90	62
Hitpass L 2022 [20]	NanoKnife^®^, AngioDynamics	CT	GA	Perc	Yes	n/a	70	n/a

GA = General anaesthesia. Perc = Percutaneous. USS = ultrasound.

### 3.5. Outcomes of IRE (Table 5)

Cardiac arrythmia occurred in nine (5%) patients. There were no fatal outcomes from cardiac arrythmia. Six (3%) bile leaks were reported including one late biliary stricture. There were two (1.1%) procedure-related deaths in 180 patients. Procedure-related haemorrhage occurred in seven (4%), with one patient requiring urgent laparotomy for control of bleeding. There were no reports of procedure-related liver failure. There were two (1%) procedure-related deaths. Information on time to local recurrence was provided by five studies and ranged from 0 months in patients with residual disease after IRE, up to 10 months. Four reports provided information on progression-free survival which ranged from 4 to 12 months. Overall survival was reported in four studies and included a 2-year OS for 62%, 61% at 24 months and an OS of 2.7 years. As patients underwent a variable number of other anti-cancer interventions both prior to and after IRE, no outcomes can be attributed solely to irreversible electroporation.

**Table 5:** Outcomes of IRE.

First Author (Year)	n	Cardiac Arrythmia	Bile Leak	Haemorrhage/ Vascular Injury	Liver Failure	Procedure-Related Mortality	Local Recurrence After IRE	PFS (Months) (95%CI)	OS
Hosein P (2014) [15]	29	2 (7%)	0	0	0	0		4 (1.4–6.6)	Median OS = Not reached. Two-year OS = 62% (37–87%)
Scheffer HJ (2014) [9]	10	1	0	0	0	0	n/a *	n/a *	n/a *
Eller A (2015) [16]	8	0	0	2 (25%)	0	0	3 months	12 (9–14) range	No mortality at 388 ± 160 days.
Beyer LP (2017) [17]	18	0	0	0	0	0	0% at 6 weeks	n/a	n/a
Frühling P (2017) [18]	16	2 (13%)	0	1 (6)	0	1 (6)	5/23 (22%) (lesions)	n/a	n/a
Cornelis FH (2020) [19]	25	0	0	0	0	0	4/29 Tumours (14%) at 4–8 weeks		15 (61%) at 24 months
Meijerink M (2021) [10]	51	4 (8%)	1 (2%)	4 (8%)	0	1 (2%)	n/a	68% (95% CI: 59–84) @ 12 months	2.7 years (95% CI: 1.6–3.8) (from IRE)
Hitpass L (2022) [20]	23	0	5 (22%) Biliary stricture	0	0	0	10 months (95% CI 8.6–11.2)	7 months (95% CI 5–9).	n/a
Total	180	9 (5)	1 (0.5)	7 (3.8)	0 (0)	2 (1.1)	-	-	-

PFS = Progression-free survival. OS = overall survival. * Patients underwent IRE followed by liver resection of the ablated tumour.

## 4. Discussion

This systematic review reports on the use of IRE for the treatment of patients with liver metastases of colorectal cancer origin. To date, much of the original literature surrounding liver-directed IRE and resultant systematic reviews has focused on the ablation of liver tumours, regardless of aetiology [21,22,23]. Whilst this is relevant in terms of the assessment of the technical feasibility, safety and procedure-related outcome of IRE, and possibly reflects the interventional radiology perspective of many of these studies, few reports give disease-specific outcome data. Therefore, it is not possible to assess the potential role of IRE in a treatment algorithm for patients with colorectal liver metastases. For example, should IRE be used as a stand-alone ablation, in conjunction with other modalities? Should it be used as an early pathway treatment or later as a salvage option? This study therefore seeks to address this information gap by focusing on the use of IRE for patients with colorectal liver metastases.

Several important limitations of this systematic review should be discussed, and the article should be read bearing these in mind. First, as much of the literature on the use of IRE for liver tumours has not been reported in a disease-specific pattern and as this study focused on reports exclusively in patients with colorectal hepatic metastases, the present report does not include all published experience on patients undergoing IRE for colorectal liver metastases. Specifically, there will be experience of IRE in patients with colorectal hepatic metastases where outcome reporting is not provided in a disease-specific pattern. Second, the reports in this series include patients at different points in their disease course and thus this review cannot draw conclusions about where IRE could be used in treatment pathways for patients with colorectal liver metastases. Specifically, some reports in this study use IRE in patients with an intact liver (with or without prior chemotherapy) whilst others use this as a later treatment in patients who have undergone hepatic resection. Third, this report represents a balance between the use of broad inclusion criteria which would (for example) potentially include treatments such as electrochemotherapy, and narrow entry requirements which result in the omission of potentially valuable reports. The MINORS criteria (Table 1) provide objective evidence of the limited data quality of the source reports. Fourth, as patients underwent a range of interventions both prior to and after IRE, it is not possible to attribute the contribution of the intervention to either progression-free or overall survival.

Having said this, what can be learnt from this paper? It is thought to be the largest collective report to date on patients undergoing IRE for colorectal liver metastases. The first point to emerge from these data is the need for more standardized and better quality of reporting. In terms of disease demographics, few studies have reported information on the site of the primary tumour, the presence or absence of extrahepatic disease, or the description of interventions prior to IRE. The use of a standardized disease-specific dataset would be of considerable value here. It seems a reasonable interpretation from the data reported here that centres favour the use of this technique for tumours close to hepatic inflow, outflow or vena cava (all sites where a heatsink would potentially compromise thermal ablation). This is clinically relevant as neither radiofrequency nor microwave ablation can be safely used in such settings [6,24]. IRE is used in reports in this series for tumours under 3 cm in size and this may be regarded as an upper limit of lesion size for this technique.

There is a substantial heterogeneity of treatments, with some reports using a combination of IRE with RFA and SIRT despite the published limited efficacy of the latter intervention for the treatment of colorectal hepatic metastases [25]. In keeping with a treatment used in the metastatic setting, systemic chemotherapy is the most frequently used pre-IRE treatment. IRE is also used in the setting of recurrent liver metastases after prior hepatectomy.

The details of the IRE procedure (Table 4) demonstrate the relative standardisation of the technique in terms of image-guided probe placement, cardiac cycle synchronisation, probe separation and treatment pulses. Cardiac cycle synchronization is critical in terms of safety when applying electrical current between electrodes placed in the liver [26]. These reports also demonstrate compliance with the Ruarus consensus [27].

The reporting of procedure-related outcomes shows two (1.1%) procedure-related deaths in 180 patients. This is likely to be regarded as an acceptable mortality rate for this procedure. The reported complication data are also within what would be regarded as acceptable limits for a liver-directed ablative treatment. It is worth bearing in mind that this reporting could have an inherent bias as it originates from experts with experience in this technique. Data on reporting outcomes are heterogeneous. As patients underwent a variable number of other anti-cancer interventions both prior to and after IRE, no progression-free or overall survival outcomes can be attributed solely to this intervention. This is a critical limitation towards the overall acceptance of IRE for colorectal hepatic metastases. Although it is accepted that a randomized evaluation remains difficult due to lack of clear comparator treatments, more information on the potential efficacy or otherwise of IRE for colorectal liver metastases could be gained from an adequate Phase II study.

A lesson learned from this overview is that it would be useful in future reports to have at least an agreed minimum dataset for reporting. This should include, in addition to demographic and tumour specific information, detail on patterns of recurrence (ablation site or new liver tumours), types of intervention and outcome.

In summary, this systematic review of IRE for patients with colorectal liver metastases shows that in over a decade of use, the reporting of the technique remains heterogenous. IRE for colorectal liver metastases can be accomplished with low procedure-related morbidity and mortality. Further prospective study is required to assess the role of IRE in the portfolio of treatments for patients with liver metastases from colorectal cancer.

## 5. Conclusions

This is a systematic review of the use of irreversible electroporation to treat patients with colorectal liver metastases. Much of the early IRE literature focused on the ablation of liver tumours without providing information on aetiology, extent of disease or prior treatments. Thus, the present article is thought to be the first systematic review to focus on IRE specifically in patients with colorectal hepatic metastases. The first learning point to emerge from these data is the heterogeneity of reporting. In particular, important aspects such as the status of the primary tumour, presence or absence of extrahepatic metastatic disease and use of prior treatments including surgery are not consistently provided. These points should be included in a minimum reporting dataset. IRE was used for tumours which were less than 3 cm in size and typically located close to either inflow or outflow structures. In this regard, the absence of thermal injury associated with electroporation may be seen as a useful and important feature and may define a future niche role for the technique. All reports describe undertaking IRE under cardiac cycle synchronization. Procedure-related morbidity is acceptable with a low-incidence of procedure-related haemorrhage and bile leak. IRE-related liver failure has, to date, not been reported in the published literature. As IRE is typically provided as part of a package of treatments including systemic chemotherapy, surgery and often other forms of ablation, it is not possible to calculate an intervention-related effect on either progression-free or overall survival. However, though the data are limited, there does appear to be a promising anti-tumour effect, and after successful IRE there are acceptable local recurrence rates. Further careful Phase II study with adequate description of disease distribution and prior intervention is required for evaluation of the role of IRE in the treatment of patients with liver metastases from colorectal cancer.

## Data Availability

No new data were created and thus there are no additional data available from this study.

## Supplementary Materials

The following supporting information can be downloaded at: https://www.mdpi.com/article/10.3390/cancers15092428/s1, Table S1: Full search strategy.
