# Supplementary material for: Irreversible Electroporation for Liver Metastases from Colorectal Cancer: A Systematic Review
Source: Cancers (Basel). 2023 Apr 24;15(9):2428. doi: 10.3390/cancers15092428 (PMC10177346; doi:10.3390/cancers15092428)
Supplement: Supplementary file 1 [file cancers-15-02428-s001.zip › cancers-2319477-supplementary.pdf]

Supplementary Material: Irreversible electroporation for liver metastases from colorectal cancer: A systematic review.

Harry VM Spiers[1,2], Francesco Lancellotti[3], Nicola de Liguori Carino[3], Sanjay Pandanaboyana[4], Adam E Frampton[5], Santhalingam Jegatheeswaran[3], Vinotha Nadarajah[6] and Ajith K Siriwardena[3*].

Supplementary Table S1: Full search strategy. * Patients underwent IRE followed by liver resection of the ablated tumour.

#	Search Term	Result
	Embase <1974 to 2023 6th January 13>	
1	exp irreversible electroporation/	1315
2	irreversible electroporation.ti,ab.	1934
3	exp colon cancer/	322851
4	colon cancer.ti,ab.	77988
5	rectum cancer.ti,ab.	267454
6	exp colorectal cancer/	946
7	colorectal cancer.ti,ab.	220224
8	exp liver metastasis/	183674
9	liver metast*.ti,ab	73307
10	1 or 2	45024
11	3 or 4	340055
12	5 or 6	267680
13	7 or 8	260385
14	9 or 10	82537
15	12 or 13 or 14 or 15	466324
16	11 and 16	24
	Ovid MEDLINE(R) <1946 to January 13th 2023>	
1	exp Electroporaion/	9356
2	irreversible electroporation.ti,ab.	953
3	exp Colonic Neoplasms/	81250
4	colon cancer*.ti,ab.	48164
5	exp Rectal Neoplasms/	52895
6	rectal cancer*.ti,ab.	24746
7	exp Colorectal Neoplasms/	231779
8	colorectal cancer*.ti,ab.	107151
9	exp Liver Neoplasms/	188863
10	liver metast*.ti,ab.	25275
11	hepatic metast*.ti,ab.	7282
12	1 or 2	9496
13	3 or 4	101082
14	5 or 6	57360
15	7 or 8	246848
16	9 or 10 or 11	196993
17	13 or 14 or 15 or 16	435529
18	12 and 17	343
	Web of Science <1900 to January 13th 2023>	
1	((ALL=(irreversible electroporation)) OR (ALL=(irreversible electroporation.ti,ab)))	2092
2	ALL=(colon OR rectum OR colorectal)	521934

3	ALL=(neoplasm OR cancer)	4528674
4	#3 AND #2	357482
5	((ALL=(liver metast*)) AND (ALL=(hepatic metast*)))	21341
6	#5 AND #4	9140
7	#6 AND #1	55
	Cochrane Trials Library <January 13th 2023>	
1	MeSH descriptor: [Electroporation] explode all trees	40
2	irreversible electroporation	75
3	colorectal liver metast*	2039
4	liver metastas*	5272
5	MeSH descriptor: [Liver Neoplasms] this term only	2678
6	MeSH descriptor: [Rectal Neoplasms] this term only	1938
7	MeSH descriptor: [Colonic Neoplasms] explode all trees	1941
8	MeSH descriptor: [Colorectal Neoplasms] explode all trees	9388
9	#1 OR #2	110
10	#3 OR #4	5547
11	#5 OR #6 OR #7 OR #8	11550
12	#10 OR #11	15984
13	#9 AND #12	9
